# Structural and functional changes of the cerebellum in temporal lobe epilepsy

**DOI:** 10.3389/fneur.2023.1213224

**Published:** 2023-08-03

**Authors:** Ge Wang, Xianghe Liu, Min Zhang, Kangrun Wang, Chaorong Liu, Yayu Chen, Wenyue Wu, Haiting Zhao, Bo Xiao, Lily Wan, Lili Long

**Affiliations:** ^1^Department of Neurology, Xiangya Hospital, Central South University, Changsha, China; ^2^National Clinical Research Center for Geriatric Disorders, Xiangya Hospital, Central South University, Changsha, China; ^3^Clinical Research Center for Epileptic Disease of Hunan Province, Central South University, Changsha, China; ^4^Department of Infectious Diseases, Xiangya Hospital, Central South University, Changsha, China; ^5^Department of Neurology, The Second Affiliated Hospital of Nanchang University, Nanchang, Jiangxi, China; ^6^Department of Anatomy and Neurobiology, Central South University Xiangya Medical School, Changsha, Hunan, China

**Keywords:** cerebellar peduncle, cerebellum, functional connectivity, multimodality MRI, temporal lobe epilepsy

## Abstract

**Aims:**

This study aimed to comprehensively explore the cerebellar structural and functional changes in temporal lobe epilepsy (TLE) and its association with clinical information.

**Methods:**

The SUIT toolbox was utilized to perform cerebellar volume and diffusion analysis. In addition, we extracted the average diffusion values of cerebellar peduncle tracts to investigate microstructure alterations. Seed-based whole-brain analysis was used to investigate cerebellar–cerebral functional connectivity (FC). Subgroup analyses were performed to identify the cerebellar participation in TLE with/without hippocampal sclerosis (HS)/focal-to-bilateral tonic–clonic seizure (FBTCS) and TLE with different lateralization.

**Results:**

TLE showed widespread gray matter atrophy in bilateral crusII, VIIb, VIIIb, left crusI, and left VIIIa. Both voxel and tract analysis observed diffusion abnormalities in cerebellar afferent peduncles. Reduced FC between the right crus II and the left parahippocampal cortex was found in TLE. Additionally, TLE showed increased FCs between left lobules VI–VIII and cortical nodes of the dorsal attention and visual networks. Across all patients, decreased FC was associated with poorer cognitive function, while increased FCs appeared to reflect compensatory effects. The cerebellar structural changes were mainly observed in HS and FBTCS subgroups and were regardless of seizure lateralization, while cerebellar–cerebral FC alterations were similar in all subgroups.

**Conclusion:**

TLE exhibited microstructural changes in the cerebellum, mainly related to HS and FBTCS. In addition, altered cerebellar–cerebral functional connectivity is associated with common cognitive alterations in TLE.

## 1. Introduction

Temporal lobe epilepsy (TLE) is the most common form of focal epilepsy, which originates from the mesial (i.e., amygdalohippocampal) or lateral regions of the temporal lobe and propagates throughout brain networks beyond the epileptic focus (1). While the cerebellum looks like a functionally separate structure, it is richly interconnected with the brainstem and/or cerebral structures and coordinates a broad range of motor and cognitive functions of brain networks (2). At present, there is a growing recognition of the importance of the cerebellar circuits and their functions in TLE.

The cerebellum is thought to interact with cerebral structures through three cerebellar peduncles: The superior cerebellar peduncle (SCP) contains fibers carrying information from the cerebellum to the cerebral cortex; the fibers of the middle cerebellar peduncle (MCP) travel from the cerebral cortex to the contralateral cerebellum *via* cerebro-ponto-cerebellar pathways; and the inferior cerebellar peduncle (ICP) links the medulla oblongata to the cerebellum, including axons from the inferior olivary nucleus as the main afferent component (3, 4). These cerebellar peduncles constitute a closed-loop cerebellar–cortical loop responsible for bidirectional interactions. Therefore, aberrant hippocampal and cortical activities in TLE might modulate the cerebellum structure and function (5), which in turn might contribute to the motor and cognitive properties of TLE.

Previous research has looked at cerebellar volumetric abnormalities in TLE (6–15). However, only two recent studies reported cerebellar substructural abnormalities with inconsistent results (16, 17). In addition, studies have not specifically focused on cerebellar diffusion features and lobule-related functional networks since functional regions of cerebellar lobules follow a gradual organization that progresses from primary (motor) to trans-modality [default mode network (DMN)], which corresponds to cerebral–cortical network organization (2). It is meant to clarify alterations in cerebellar sublobules and their connections with the cortex in TLE. The main objective of the present study focuses on three points: (1) Do substructures of the cerebellar gray matter have different volumes between TLE and healthy controls (HCs)? (2) Do cerebellar afferent and efferent tracts have abnormal structural features in TLE? (3) Do functional and structural alterations in the cerebellar lobules relate to clinical manifestations? We adopted multimodality MRI and the spatially unbiased infratentorial (SUIT) toolbox to provide an optimized and fine-grained exploration of cerebellar structural and related functional alterations associated with TLE.

## 2. Materials and methods

### 2.1. Participants

All subjects were collected from Xiangya Hospital, Central South University, including 73 TLE patients and 74 demographically matched HCs. TLE was confirmed by two certified neurologists in epilepsy based on the criteria defined by the International League Against Epilepsy (18). Seizure lateralization and focal-to-bilateral tonic–clonic seizure (FBTCS) of each TLE patient were determined by assessing seizure semiology, epileptiform discharges on ictal and interictal EEG (left TLE [LTLE]: right TLE [RTLE] = 42:31) (TLE-FBTCS: TLE-non-FBTCS = 54:19). FBTCS frequency was estimated for a period of 12 months before scanning. Two experienced neuroimagers visually assessed the MRI images (T2-weighted imaging, T2 fluid-attenuated inversion recovery, and three-dimensional T1 sequences) to identify hippocampal sclerosis (HS) (TLE-HS: TLE-non-HS = 36:37) (19). Except for the presence of HS, all participants demonstrated normal MRI and had no history of neurological/psychiatric illness.

### 2.2. Neuropsychological assessment

Neuropsychological tests included the following: (1) global cognitive status: Montreal Cognitive Assessment (MoCA); (2) executive functions and attention: Trail-Making Test part-A (TMTA) and part-B (TMTB); (3) short-term and working memory: Digit Span Test Backward (DS-B) and Forward (DS-F); (4) spatial visualization ability: Block Design Test (BDT).

### 2.3. Magnetic resonance data acquisition

Multimodality MRI for all subjects was acquired on a 3T General Electric (Signa HDx, USA) scanner with a 32-channel phased-array head coil. All subjects were instructed to keep their eyes closed and stay awake during scanning.

High-resolution 3D brain anatomical images were obtained using a T1-weighted MP-RAGE sequence [TR = 7,792 ms, TE = 2.984 ms, FOV = 256 × 256 mm (2), matrix = 256 × 256, number of slices: 188, slice thickness: 1 mm, FA: 7 degrees, voxel size: 1 × 1 × 1 mm (3)]. Diffusion-weighted images were obtained using a single-shot echo-planar imaging sequence [TR = 12,000 ms, TE = 76.9 ms, FA= 90 degrees, number of slices: 55, slice thickness: 3 mm, FOV = 256 × 256 mm (2), matrix = 128 × 128, voxel size: 2 × 2 × 3 mm (3), b-value: 1,000 s/mm (2), 32 non-collinear gradient directions]. Resting-state functional images (rs-fMRI) were collected using a gradient-echo echo-planar imaging sequence [TR/TE = 2,000/30 ms, thickness = 4 mm, matrix size = 64 × 64, FOV=220 × 220 mm (2), FA= 90 degrees, slices = 32].

### 2.4. Data processing

#### 2.4.1. MR data

##### 2.4.1.1. Cerebellar volume analysis

The optimized voxel-based morphometry of infratentorial structures was performed using the spatially unbiased infratentorial template (SUIT) toolbox implemented in Statistical Parametric Mapping software, version 12 (SPM12, http://www.fil.ion.ucl.ac.uk/spm) (20). The process briefly includes visually checking, localizing anterior commissure, cerebellum and brainstem segmentation, manual corrections, normalization to SUIT template, and Jacobian modulation. A 4 mm reasonable FWHM Gaussian kernel was used for smoothing (21, 22). Two-sample *t*-tests were performed in SPM12 for voxel-based analyses, with control for sex, age, and total intracranial volume (TIV) calculated using the CAT12 toolbox (23). The results were corrected within the cerebellar mask using FDR (*q* < 0.05, voxel level; cluster ≥ 10). In addition, a ROI analysis of cerebellum subfields was performed using IBM SPSS Statistics 24, employing a two-sided *t*-test with age, sex, and TIV as covariates. Significance was determined at false discovery rate (FDR) *q*-values < 0.05.

##### 2.4.1.2. Cerebellar DTI analysis

DTI scans were preprocessed and analyzed using the FMRIB's Diffusion Toolbox in FSL software (https://fsl.fmrib.ox.ac.uk/fsl/fslwiki/) (24). The original data were corrected for head motion and eddy currents using the eddy correct command by applying affine registration to the first b = 0 volume. The fractional anisotropy (FA), axial diffusivity (AD), mean diffusivity (MD), and radial diffusivity (RD) whole-brain maps for each DTI image were calculated by DTIFIT and then co-registered to the subject's T1-weighted image. The cerebellum and brainstem were segmented and spatially normalized to the SUIT template. The following preprocess includes Jacobian modulation and smoothness (4 mm FWHM). Voxel-based DTI analysis, with sex and age as covariates, was conducted to compare group differences in diffusion characteristics. The results were corrected within the cerebellar mask using a common threshold of FDR corrected *q* < 0.05 at the voxel level and cluster ≥ 10. Significant clusters were identified using the SUIT atlas and Duvernoy's Atlas of the Human Brainstem and Cerebellum (25). Average diffusion values of specific cerebellar peduncles (SCP, MCP, and ICP) were extracted from the ICBM-DTI-81 atlas for further comparison (26). Significant differences were determined according to a *p*-value of < 0.0083 (Bonferroni corrected for multiple comparisons, *p* < 0.05) (27).

##### 2.4.1.3. Functional connectivity analysis

Rs-fMRI data were commonly preprocessed using SPM12 (https://www.fil.ion.ucl.ac.uk/spm) with the following steps: removing the first ten volumes of data, slice timing and motion correction, normalization with an EPI template, spatial smoothing (6 mm FWHM), regression of nuisance variables (head motion, average ventricular and white matter signals), linear detrending, and band-pass temporal filtering (0.01–0.08 Hz). Global signal regression of the gray matter voxels was not included in the denoising process because this was known to introduce spurious anticorrelations between large networks (28), and the global signal itself contained neural information (29–31). Whole-brain voxel-wise FC analysis was performed, and the connectivity maps were converted to z-scores using Fisher's r-to-z transform. Two-sample *t*-tests with sex and age as covariates were performed. The resultant T-maps were corrected using the Gaussian random field (GRF) method for whole-brain analysis (voxel-wise *p* < 0.001 and cluster ≥50, two-tailed, GRF-corrected) (32).

#### 2.4.2. Correlation analysis between abnormal imaging measures and clinical features

We conducted ROI-wise correlation analyses to examine the relationship between various clinical variables (including age at onset, disease duration, FBTCS frequency, the number of antiseizure medications, and neuropsychological scores) and abnormal imaging findings in GM, DTI, and FC. Partial correlations were performed with age and gender as covariates (15, 22). The statistical significance of these correlations was assessed using FDR correction. Additionally, we also reported the results with an exploratory *p*-value threshold of 0.05. For voxel-wise correlation analyses, we explored the association between these clinical features and clusters (GM and WM) that exhibited significant group differences. The exploratory threshold for voxel-wise analyses was set at a *p*-value of < 0.001, uncorrected, with a minimum cluster size of 10 (33).

#### 2.4.3. Subgroup analysis

In order to determine the cerebellar involvement in different subgroups of TLE, we divided the TLE patients based on seizure lateralization, the presence or absence of HS, and the presence or absence of FBTCS. Statistical comparisons were then performed between control participants and each subgroup of TLE patients, including those with left or right lateralization, with or without HS, and with or without FBTCS. For voxel-based multiple comparisons, we employed a threshold of FWE correction at a *p*-value of < 0.05 and cluster ≥ 10. In addition, for ROI-based multiple comparisons, *post-hoc* Bonferroni analysis (*p* < 0.05) was applied.

Given that the sample size of the TLE-non-FBTCS group was relatively smaller compared to the TLE-FBTCS and healthy control groups, sensitivity analyses were repeated using permutation-based correction implemented in Statistical non-Parametric Mapping software to ensure that the results were not dependent on the selected statistical approach (http://warwick.ac.uk/snpm).

## 3. Results

### 3.1. Demographic and clinical data

There were no significant differences in gender and age between the control and TLE group. Controls outperformed patients on all neuropsychological assessments (*p* < 0.001). Clinical details of the TLE group are listed in Table 1. All recruited patients were undergoing antiseizure medication at the time of imaging. Patients were treated as follows: oxcarbazepine (n = 54), mean daily dose: 986 ± 260 mg; valproic acid (28), 1,059 ± 283 mg; levetiracetam (19), 1,158 ± 291 mg; lamotrigine (14), 200 ± 65 mg; topiramate (8), 187 ± 23 mg; and carbamazepine (5), 600 ± 255 mg. Demographic and clinical details are listed in Table 1. We also provided detailed data on the subgroups for potential utility in Supplementary Table 1.

**Table 1:** Demographic and clinical information for TLE and HC.

**Characteristic**	**TLE**	**HC**	***P*-value**
**Number**	73	74	
**Age**, years, mean (SD)	31.2 (12.0)	30.7 (11.3)	0.83^a^
**Sex**, male/female	32/41	39/35	0.28^b^
**Duration**, years, mean (SD)	9.1 (8.0)		
**AOO**, years, mean (SD)	21.9 (12.8)		
**Febrile convulsion history**	6		
**Seizures**			
Without FBTCS	19		
With FBTCS	54		
**FBTCS frequency**, per year, mean (SD)	9 (15)		
**FIAS frequency**			
≤ 1 per month	27		
2–4 times per month	11		
>4 times per month	35		
**HS**			
With HS	36		
Without HS	37		
**Number of ASMs**			
1	25		
2	41		
3	7		
**Neuropsychology**			
MoCA, mean (SD)	22 (5)	28 (3)	< 0.001^a*^
DS-F, mean (SD)	7 (1)	8 (2)	< 0.001^a*^
DS-B, mean (SD)	4 (1)	6 (2)	< 0.001^a*^
BDT, mean (SD)	32 (10)	38 (10)	< 0.001^a*^
TMTA, mean (SD)	52 (24)	40 (16)	< 0.001^a*^
TMTB, mean (SD)	139 (73)	87 (51)	< 0.001^a*^

^a^Obtained by two-sample two-tailed t-test.

^b^Obtained by chi-square analysis.

^*^p < 0.05.

AOO, age at onset; ASM, antiseizure medication; BDT, block design test; FIAS, focal impaired awareness seizure; DS-B, Digit Span-Backward; DS-F, Digit Span-Forward; HC, healthy controls; HS, hippocampal sclerosis; MoCA, Montreal Cognitive Assessment; FBTCS, focal-to-bilateral tonic–clonic seizure; TMTA, Trail-Making Test part-A; TMTB, Trail-Making Test part-B.

### 3.2. Cerebellar volume results

**Figure 1:**
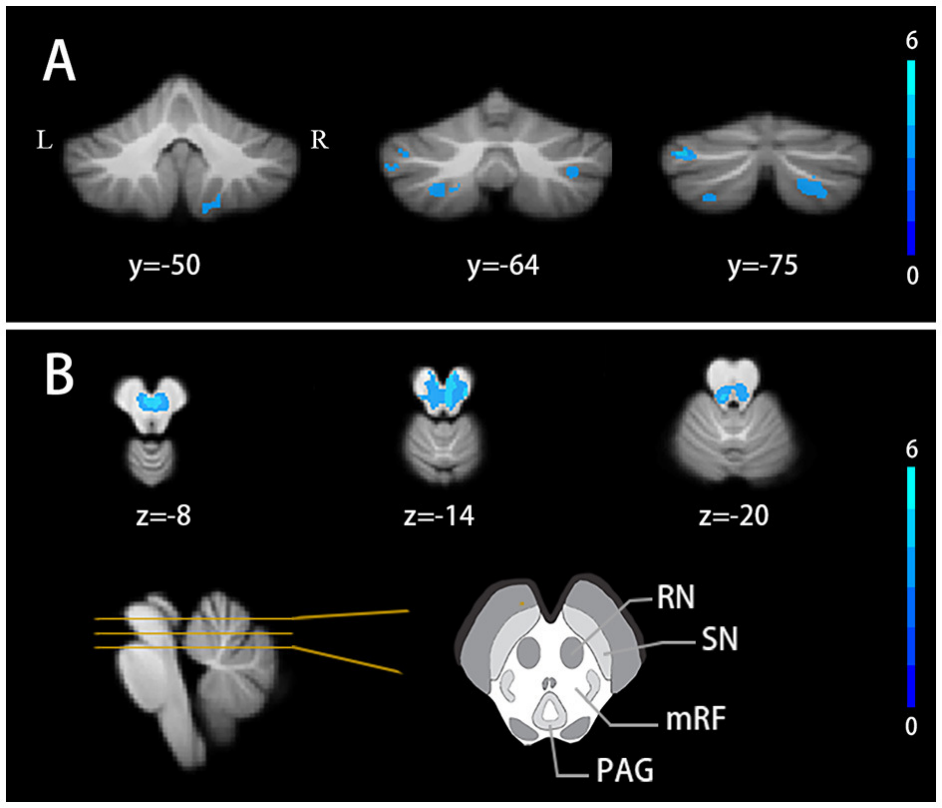
Cerebellar volume alterations in TLE patients **(A)** Cerebellar lobules atrophy in TLE patients. **(B)** Gray matter nuclei atrophy in TLE patients. FDR correction q < 0.05, cluster ≥ 10. Results are projected on the SUIT atlas. RN, red nucleus; SN, substantia nigra; mRF, mesencephalic reticular formation; PAG, periaqueductal gray.

The SUIT analysis revealed widespread GM atrophy. Specifically, both voxel-wise and ROI-wise analyses revealed significant volumetric reductions in bilateral crusII, VIIb, VIIIb, left crusI, and left VIIIa in TLE patients (FDR correction *q* < 0.05, cluster ≥ 10) (Figure 1A, Supplementary Tables 2, 3). TLE patients also showed a volume decrease in the midbrain with the peak MNI coordinates at the right red nucleus (peak MNI coordinates: 4,−18,−12). Other atrophy structures include the left red nucleus, bilateral mesencephalic reticular formation, and periaqueductal gray (FDR correction *q* < 0.05, cluster ≥ 10) (Figure 1B).

### 3.3. Cerebellar diffusion results

**Figure 2:**
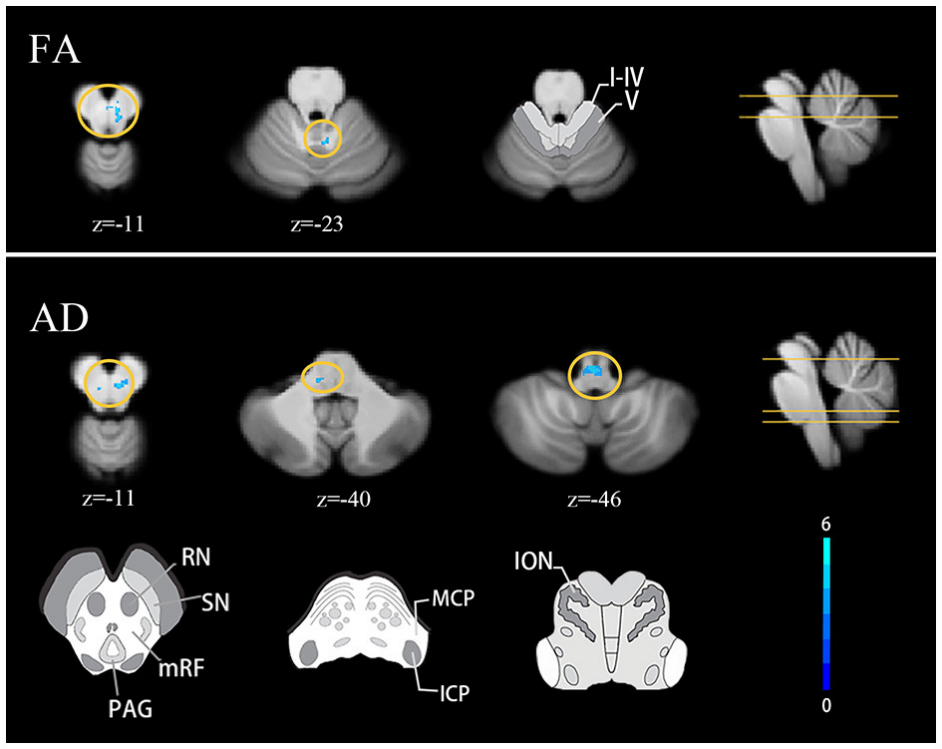
Cerebellar diffusion alterations in TLE patients FDR correction q < 0.05, cluster ≥ 10. Results are projected on the SUIT atlas. MCP, middle cerebellar peduncle; ICP, inferior cerebellar peduncle; ION, inferior olivary nucleus.

Compared with healthy controls, the TLE group showed decreased FA at the right red nucleus and mesencephalic reticular formation and AD at the right red nucleus and bilateral mesencephalic reticular formation. Decreased FA was also found around the right lobule IV and V compared with HCs (FDR correction *q* < 0.05, cluster ≥ 10) (Figure 2).

Both voxel and tract analyses revealed lower AD in cerebellar afferent tracts of the TLE group. Voxel-based analysis showed decreased AD at left MCP and left inferior olivary nucleus (FDR correction *q* < 0.05, cluster ≥ 10) (Figure 2). Tract analysis showed lower average AD in left MCP and bilateral ICP (FWE-corrected *p* < 0.05) (Table 2).

**Table 2:** Diffusion characteristics for bilateral cerebellar peduncles in the TLE and HC.

	**L-SCP**	**R-SCP**	**L-MCP**	**R-MCP**	**L-ICP**	**R-ICP**
**FA**						
TLE	0.208 ± 0.032	0.341 ± 0.055	0.460 ± 0.102	0.460 ± 0.117	0.275 ± 0.059	0.232 ± 0.045
HC	0.213 ± 0.033	0.344 ± 0.0633	0.432 ± 0.126	0.431 ± 0.134	0.275 ± 0.051	0.233 ± 0.053
*P*-value	0.417	0.729	0.152	0.167	0.981	0.969
**AD**						
TLE	0.532 ± 0.085	0.787 ± 0.098	1.109 ± 0.261	1.102 ± 0.163	0.882 ± 0.181	0.743 ± 0.144
HC	0.525 ± 0.098	0.819 ± 0.098	1.275 ± 0.420	1.199 ± 0.275	0.989 ± 0.281	0.890 ± 0.348
*P*-value	0.653	0.0568	0.006^***^	0.012	0.008^***^	0.001^***^
**MD**						
TLE	0.323 ± 0.047	0.472 ± 0.050	0.683 ± 0.246	0.656 ± 0.166	0.605 ± 0.170	0.502 ± 0.128
HC	0.326 ± 0.054	0.496 ± 0.083	0.815 ± 0.405	0.731 ± 0.278	0.685 ± 0.281	0.618 ± 0.346
*P*-value	0.718	0.037	0.021	0.055	0.043	0.009
**RD**						
TLE	0.219 ± 0.042	0.314 ± 0.062	0.462 ± 0.227	0.454 ± 0.234	0.464 ± 0.150	0.387 ± 0.121
HC	0.222 ± 0.048	0.327 ± 0.084	0.546 ± 0.334	0.488 ± 0.263	0.502 ± 0.192	0.452 ± 0.272
*P*-value	0.664	0.275	0.085	0.419	0.190	0.070

^***^Statistical significance was set at p < 0.0083 (FWE-corrected p < 0.05).

The MD, RD, and AD metrics are shown as 10–3, and FA is shown as the actual value.

Since 3 mm FWHM smooth for cerebellar analysis might prompt the exploration of more subtle cerebellar changes and has been applied in some research, we also used a 3 mm smoothing kernel and found similar results.

### 3.4. Functional connectivity results

**Figure 3:**
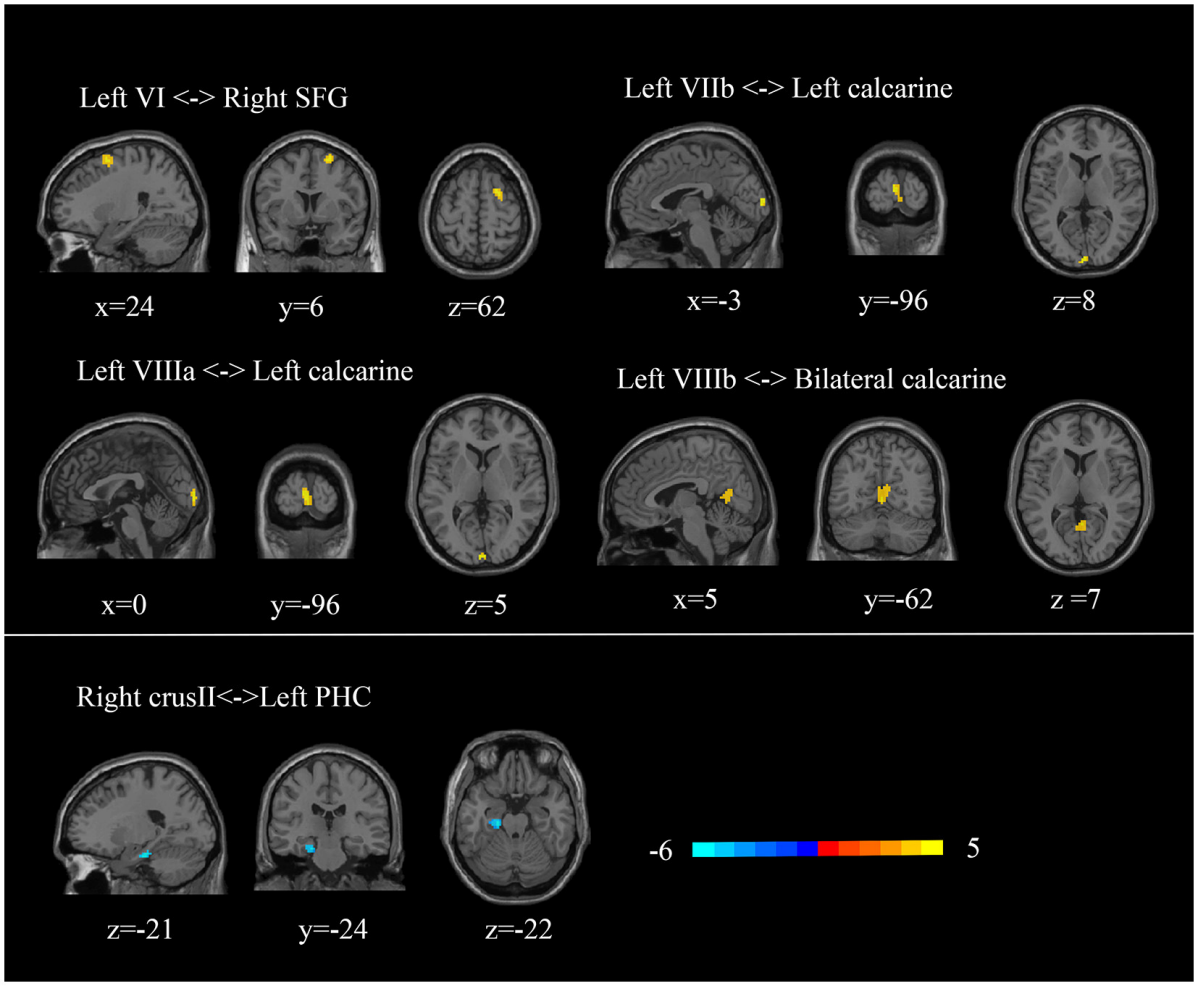
Abnormal cerebellar functional connectivity in TLE patients. SFG, superior frontal gyrus; PHC, parahippocampal cortex.

Since atrophy regions locate in cerebellar triple non-motor (lobules VI/Crus I, Crus II/VIIb, and IX/X) representation (2), we included these lobules as ROIs to identify potential functional alterations. We found a decreased FC between the right crus II and the left parahippocampal cortex in TLE patients (*p* < 0.0005, cluster ≥ 50, GRF-corrected) (Figure 3). TLE patients also showed increased FC between the left lobule VI and the right superior frontal gyrus, the left VIIb and the left calcarine, the left VIIIa and the left calcarine, and the left VIIIb and the bilateral calcarine (*p* < 0.0005, cluster ≥ 50, GRF-corrected) (Figure 3). Through ICA, we identified that the superior frontal cortex was involved in the dorsal attention network, and the calcarine region was involved in the dorsal attention and visual networks (see Supplementary Figure 1) (34, 35).

### 3.5. Correlation analysis results

#### 3.5.1. Correlation of abnormal gray matter volume with clinical variables

**Figure 4:**
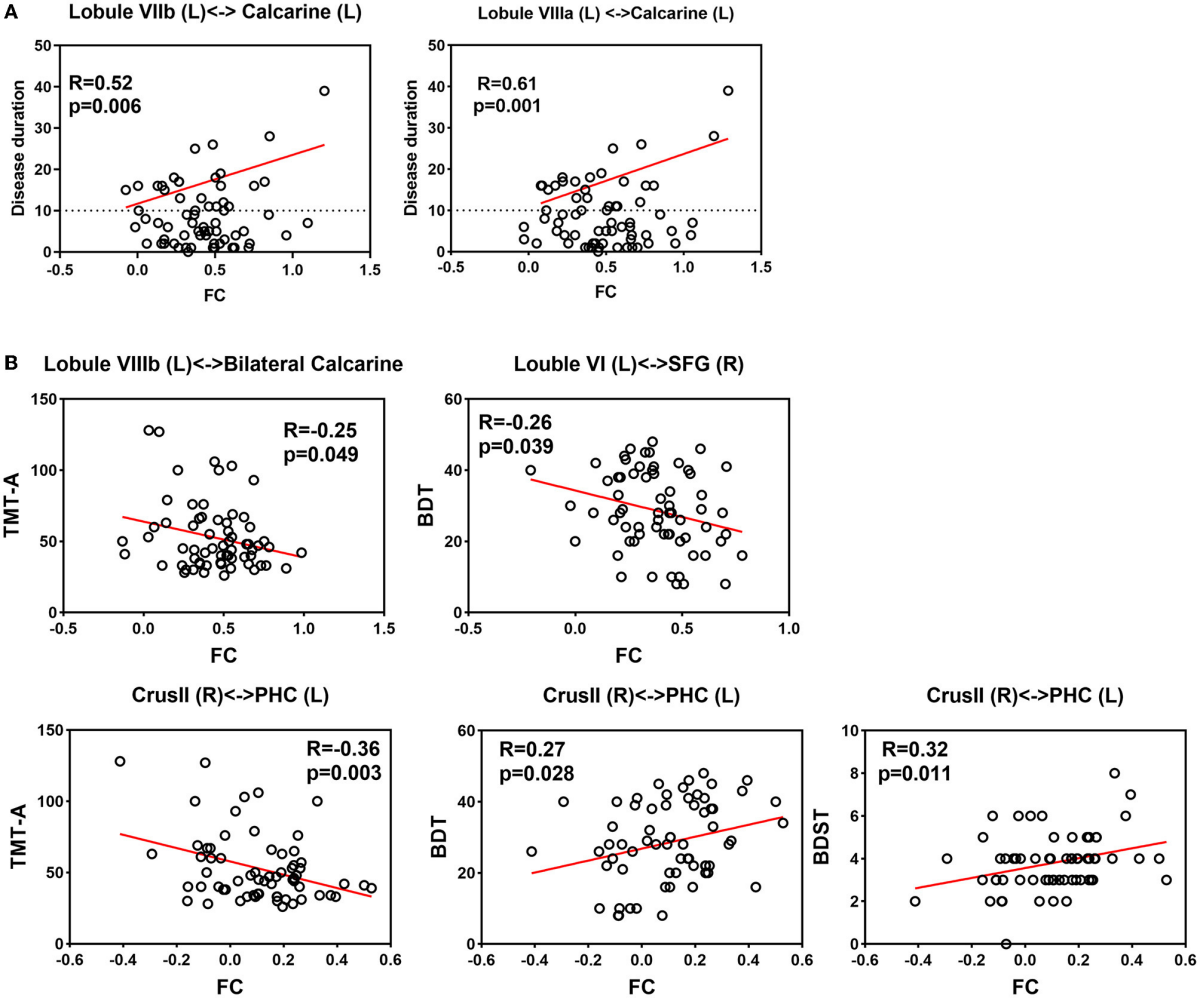
Correlation between clinical assessments and altered functional connectivity **(A)** Correlation between disease duration and increased FC. **(B)** Correlation between neuropsychological assessment and altered FC.

Our findings did not reveal any significant correlations between altered cerebellar GMV and clinical variables in either the voxel-wise or ROI-wise analyses. There was a tendency for a negative correlation between the volume of cerebellar lobe VIII and the frequency of GTCS (R = 0.43, p = −0.281). Moreover, a negative association trend was observed between TMTB and decreasing volume in the cerebellar substructures (left crusI: R = −0.298, *p* = 0.030; right crus II: R = −0.310, *p* = 0.024; left VIIIa: R = −0.326, *p* = 0.017; left VIIIb: R = −0.294, *p* = 0.033). Correlation coefficients are listed in Supplementary Table 4, Figure 4A. These correlations should be interpreted with caution, and further studies are needed to confirm and interpret these findings accurately.

#### 3.5.2.Correlation of DTI measures with clinical variables

We did not find any significant voxel-wise or ROI-wise correlation between abnormal WM measures and the age of onset, duration of illness, and the number of antiseizure medications, respectively. Correlation coefficients are listed in Supplementary Table 4.

#### 3.5.3. Correlation of altered FC results with clinical variables

For correlation analyses of epilepsy duration, our intertest interval was ≥10 years. Our findings indicated a significant positive linear relationship between disease duration exceeding 10 years and the FC between the left lobule VIIIa and the left calcarine (R = 0.61, *p* = 0.001). Additionally, there was a suggestive trend of correlation between disease duration exceeding 10 years and FC between the left lobule VIIb and the left calcarine (R = 0.43, *p* = 0.034).

Regarding neuropsychological assessments, the decreased FC between the right crus II and the left parahippocampal gyrus showed a significant correlation with TMTA completion time (R = −0.36, *p* = 0.003). We also observed a positive correlation between decreased FC (right crus II and left parahippocampal gyrus) and BDT scores (R = 0.33, *p* = 0.009), as well as DS-B scores (R = 0.26, *p* = 0.043). Additionally, there were negative correlations between increased FC and BDT scores (left lobule VI and right superior frontal gyrus: R = −0.28, *p* = 0.028; left VIIIb and bilateral calcarine: R = −0.28, *p* = 0.028). However, these correlations are statistically insignificant after FDR correction. It is worth noting that further studies are necessary to confirm the significance of these correlations and interpret them appropriately. Relationships between altered FCs and clinical features are shown in Supplementary Table 4, Figure 4B.

No significant correlation was detected between altered FC features and the age of onset, FBTCS frequency, or the number of antiseizure medications, respectively.

### 3.6. Subgroup analysis results

#### 3.6.1. Subgroup analysis for LTLE and RTLE

Structural and functional analyses between LTLE/RTLE and HC roughly replicated the results in whole group comparison (voxel-based multiple comparisons: FWE *p* < 0.05, cluster ≥ 10; ROI-based multiple comparisons: *p* < 0.05, *post-hoc* Bonferroni test) (See Supplementary Figures 2, 3, Supplementary Tables 5, 6). We did not find significant differences between LTLE and RTLE subgroups, but LTLE patients appear to have greater degrees of diffusion changes.

#### 3.6.2. Subgroup analysis for TLE-HS and TLE-non-HS

Compared to controls, TLE patients with HS showed similar volume, diffusion, and FC alterations as the total TLE group (voxel-based multiple comparisons: FWE *P* < 0.05, cluster ≥ 10; ROI-based multiple comparisons: *P* < 0.05, *post-hoc* Bonferroni test). For TLE-non-HS patients, FA and FC results roughly replicated the results in the total TLE group. Nonetheless, *post-hoc* analysis suggested no cerebellar volume atrophy and only slight AD changes in TLE-non-HS patients.

For comparisons between TLE-HS and TLE-non-HS, we observed significant FA reduction at the left inferior olivary nucleus in TLE-HS patients (FWE *P* < 0.05, cluster ≥ 10). The TLE-HS group also showed trends for decreased cerebellar GM volume and AD value (*P* < 0.0025 at the voxel level and cluster ≥ 10) (36) (See Supplementary Figures 4, 5, Supplementary Tables 7, 8).

#### 3.6.3. Subgroup analysis for TLE-FBTCS and TLE-non-FBTCS

Compared to controls, TLE patients with FBTCS showed similar cerebellar atrophy, diffusion, and FC alterations as the total TLE group (voxel-based multiple comparisons: FWE *p* < 0.05, cluster ≥ 10; ROI-based multiple comparisons: *p* < 0.05, *post-hoc* Bonferroni HSD test). FC results in the TLE-non-FBTCS group roughly replicated the results in the total TLE group. Nevertheless, there was little structural changes in TLE-non-FBTCS compared to HC.

For comparisons between TLE-FBTCS and TLE-non-FBTCS, significant cerebellar and brainstem atrophy was found in the TLE-FBTCS subgroup (FWE *p* < 0.05, cluster ≥ 10) (See Supplementary Figures 6–9, Supplementary Tables 9, 10).

## 4. Discussion

The present study observed widespread cerebellar atrophy and decreased diffusion properties in TLE, suggesting microstructure changes in the cerebellum and major tracts linking the cerebellum and extracerebellar regions. We also performed subgroup analysis. Alterations of cerebellar afferent tracts and the cerebellum are predominant in TLE-HS and TLE-FBTCS subgroups, suggesting that HS and FBTCS may participate in cerebellar structural damages. Moreover, we observed altered cerebellar-cortical FCs in TLE, which are related to disease duration and neuropsychological performances.

### 4.1. Cerebellar volume and diffusion abnormalities in TLE

In the present study, we adopted an optimized toolbox for infratentorial structures and investigated atrophy in specific cerebellar sublobules in TLE. Cerebellar volume alterations in TLE have been investigated by previous studies [total cerebellar (10), cerebellar gray and white matter (15), cerebellar hemispheres (6, 11, 12, 14), and main cerebellar lobes (7, 13, 16, 17)]. Consistent with most studies, the atrophy in our study is located at the posterior cerebellar lobe, including bilateral crusII, VIIb, VIIIb, left crusI, and left VIIIa. Moreover, we first focused on cerebellar diffusion features and observed that TLEs exhibited decreased FA around the right lobule IV and V. The mechanism of cerebellar atrophy in TLE remains unclear. Several hypotheses have been proposed: long duration of epilepsy, hypoxia during FBTCS, toxicity from antiseizure medications (particularly phenytoin), and pre-existing susceptible structures (15, 37). Subgroup and correlation analyses in the present study preliminarily revealed the mechanism.

#### 4.1.1. Absence of lateralized abnormalities in cerebellar structural changes

The present study investigated cerebellar abnormalities in left vs. right TLE and found no lateralized gray or white matter tissue abnormalities in the cerebellum. Both LTLE and RTLE groups exhibited bilateral cerebellar atrophy, with a greater volume reduction observed in the contralateral gray and white matter. These findings corroborate the previous reports that despite the presence of chronic unilateral TLE, the impact on the structure of the cerebellum is symmetric (6, 9, 12, 13). Additionally, our voxel- and ROI-wise results suggested that LTLE patients appear to have greater degrees of white matter diffusion alterations. This is consistent with previous research and might result from brain asymmetry (38). Further confirmation with larger sample sizes is warranted to verify these findings.

#### 4.1.2. Association between cerebellar atrophy and HS

In *post-hoc* analysis between TLE-HS and controls, we observed significant cerebellar atrophy in patients, including the left crus I, VIIb, VIIIa, VIIIb, and right crus II. Nonetheless, no trend of difference was observed in TLE-non-HS compared to controls. Previous studies also supported this finding. Alvim et al. focused on seizure-free TLE-HS/TLE-non-HS patients and only found cerebellar atrophy in patients with HS (39). In addition, Park et al. demonstrated that there were already volume reductions in the cerebellum in patients with newly diagnosed TLE (15). In our study, since there were no significant differences in age of onset, disease duration, number of ASMs, FBTCS frequency, and febrile convulsion history between the two subgroups, the substantial cerebellar atrophy might be strongly associated with HS and might precede the onset of epilepsy in some individuals. Currently, there is evidence supporting that the disruption of hippocampal integrity might also precede seizure onset (40). Given the potential direct and indirect pathways connecting the hippocampus and cerebellum, as well as the modulation of cerebellar activity by hippocampal epileptiform activity (41, 42), it is plausible that subtle hippocampal alterations in TLE could influence cerebellar changes prior to seizure onset through potential hippocampal–cerebellar connections.

#### 4.1.3. Relationship between cerebellar structural changes and FBTCS

Our study was the first to compare structural measures of cerebellum and brainstem among TLE-FBTCS, TLE-non-FBTCS, and HC. Widespread volume reduction and decreased FA around right lobule IV and V were found in TLE-FBTCS compared to HC. FA reflects the size, number, and coherence of myelinated axons, which is highly sensitive to microstructural alterations (43). The decreased FA suggests that TLE-associated cerebellar microstructure alterations also occurred near the cerebellar motor areas (2). In addition, we observed volumetric and diffusional abnormalities in the brainstem of TLE-FBTCS patients, including decreased GM volume, FA, and AD in red nucleus and mesencephalic reticular formation and volume atrophy in periaqueductal gray. Decreased FA and AD suggest more subtle changes in both axonal myelination and cytoarchitecture (27, 44–46), which may result from the loss of fiber integrity, axonal damage, or the destruction of intracellular compartments. In addition, the reduced GM volume in TLEs may result from atrophy of neurons or glia or synaptic loss (47). These microstructural changes were in TLE-FBTCS rather than the TLE-non-FBTCS subgroup, suggesting the effects of seizures propagating might cause microstructural changes in the brainstem and the cerebellum.

The significant cerebellar atrophy observed in TLE-FBTCS, when considering connectivity to cortical structures, could potentially be attributed to excitotoxic processes triggered by the excessive activation of pontine or olivary projections (48). This is a process wellknown in the neurotoxicity field and presumably operates similarly with hypoxia during FBTCS events, which readily kill Purkinje neurons (48). In the present study, we observed that TLE-FBTCS patients had altered AD in ICP and volume in pons. The damage to these structures, which contain decussating cerebro-ponto-cerebellar and olivo-cerebellar fibers, may be associated with excitotoxic effects. Notably, the lower AD might be attributed to axonal damage, which creates barriers that hinder water diffusion (e.g., cellular debris and microtubule disarrangement) (49). Previously, Szabó et al. observed a correlation of cerebellar asymmetries with FBTCS frequency, which might support the involvement of the decussating cerebellar tracts (11). Our study further supports this finding by observing the most significant atrophy in the contralateral cerebellar hemisphere to the side of the epileptic focus. In addition, we found no lateralized cerebellar abnormalities when comparing LTLE and RTLE groups, which might result from the generalization and bilateral excitotoxicity.

Collectively, current results suggest that HS-related pre-existing susceptible cerebellar–hippocampal pathways and FBTCS-related excitotoxic processes might be the underlying pathologic mechanisms for cerebellar alterations.

### 4.2. Cerebellar–cerebral functional connections in TLE and the relationship with cognition

According to the double motor (lobules I-VI and VIII) and triple non-motor (lobules VI/Crus I, Crus II/VIIB and IX/X) representation (2), the widespread gray matter loss in TLE mainly represents non-motor processing dysfunctions. We found decreased FC between the right crus II and the left parahippocampal gyrus in TLE. Dysfunctions of the parahippocampal gyrus play an important role in the expression of cognitive deficits associated with TLE (50). Correlation analyses in our study indicated that the decreased FC was associated with poorer performances in tasks assessing executive functions (TMTA), attention and spatial visualization ability (BDT), and short-term and working memory (DS-B).

In addition, the present study observed altered functional connectivity between cerebellar lobules and cortical nodes within dorsal attention and visual networks. The dorsal attention network and visual network are strongly correlated during rest and spatial attention (51). Previous studies demonstrated that the cerebellar subdivision (mainly lobules VI, VII, and VIII) exhibits strong intrinsic functional connectivity with the cortical dorsal attention and visual networks (52, 53). In the present study, an obvious enhancement in FC was found between the left cerebellar lobules VI, VIIb, VIIIa, and VIIIb and cortical nodes in the dorsal attention and visual network. We found that FC increases linearly as the duration of disease increases after 10 years, which may be responsible for the progressive memory impairments associated with chronic duration. A negative correlation with BDT scores might indicate damaged visuospatial attention. In addition, these functional cerebellar–cerebral alterations keep similar in TLE subgroups, suggesting that compensation is a common issue in TLE patients (54).

### 4.3. Limitations

Chronic antiseizure medication may affect the cerebellar structure and function in TLE. Although patients in our study did not take phenytoin, medications such as valproate, oxcarbazepine, topiramate, lamotrigine, and levetiracetam were widely used, which might affect the cerebellum. The effect of the antiseizure medication is hard to evaluate since the type and frequency of medication were adjusted according to the seizure symptoms. We performed correlation analysis and observed that imaging measures were independent of the number of medications. Future comprehensive prospective longitudinal studies would provide further information on the relationship between seizure burden and severity and cerebellar damage.

## 5. Conclusion

In summary, this study revealed abnormal cerebellar structural and functional abnormalities in TLE patients, which complements previous research and furthers the understanding of the cerebellum in TLE networks. We provided evidence that alterations of cerebellar volume and diffusion features mainly result from HS-related pre-existing susceptible pathways and FBTCS-related excitotoxic processes mediating by MCP and ICP. In addition, we suggested that the cerebellum might be involved in cognitive alterations in TLE patients.

## Data availability statement

The raw data supporting the conclusions of this article will be made available by the authors, without undue reservation.

## Ethics statement

The studies involving human participants were reviewed and approved by Ethics Committee of Xiangya Hospital. Written informed consent to participate in this study was provided by the participants' legal guardian/next of kin.

## Author contributions

GW, XL, and MZ were responsible for the conception and design of the study, the acquisition and analysis of data, the functional connectivity analysis, manuscript drafting, and revising. KW and CL were responsible for the acquisition and analysis of data. YC was responsible for the setting of parameters of the task and the acquisition and of data. WW and HZ were responsible for the acquisition and of data. BX designed and conceptualized study. LL and LW designed and conceptualized study and revised the final manuscript. All authors contributed to the article and approved the submitted version.
