# Supplementary material for: Structural and functional changes of the cerebellum in temporal lobe epilepsy
Source: Front Neurol. 2023 Aug 3;14:1213224. doi: 10.3389/fneur.2023.1213224 (PMC10435757; doi:10.3389/fneur.2023.1213224)
Supplement: Supplementary file 1 [file Data_Sheet_1.pdf]

Supplementary Material

Article Title

Ge Wang [1,2,3], Xianghe Liu [1,2,3], Min Zhang[1,2,3], Kangrun Wang [1,2,3], Chaorong Liu [1,2,3], Yayu Chen [1,2,4], Wenyue Wu [1,5], Haiting Zhao [1,2,3], Bo Xiao [1,2,3], Lily Wan [6] *, Lili Long[1,2,3] *

*** Correspondence:**

Lili Long：longlili1982@126.com

Lily Wan：wanll1203@csu.edu.cn

1 Demographic and clinical data for TLE subgroups

Supplementary Table 1: Demographic and clinical data for TLE subgroups

	TLE_L	TLE_R	TLE-HS	TLE-nonHS	TLE-SGS	TLE-nonSGS	Comparison		
							LTLE, RTLE, HC	TLE-HS, TLE-nonHS, HC	TLE-SGS, TLE-nonSGS, HC
**Number**	42	31	36	37	54	19	-	-	-
**Age**, years, mean (SD)	30.8 (9.6)	31.7 (14.7)	31.8 (12.0)	30.5 (12.1)	31.8 (12.7)	29.3 (9.8)	0.93[b]	0.87[b]	0.70[b]
**Sex**, male/female	21/21	11/20	16/20	16/21	25/29	7/12	0.26[c]	0.56[c]	0.46[c]
**Duration**, years, mean (SD)	9.5 (8.4)	8.6 (7.4)	9.9 (7.0)	8.4 (8.9)	8.9 (7.7)	9.7 (9.0)	0.64[a]	0.43[a]	0.71[a]
**AOO**, years, mean (SD)	21.3 (10.6)	22.7 (15.5)	21.5 (13.0)	22.2 (12.9)	22.7 (13.1)	19.6 (12.2)	0.64[a]	0.83[a]	0.38[a]
**Febrile convulsion history**	2	4	4	2	3	3	0.39[c]	0.43[c]	0.178[c]
**Seizures**									
Without SGS	12	7	9	10	0	19	0.56[c]	0.84[c]	-
With SGS	30	24	27	27	54	0			
**SGS frequency**, mean (SD)	10 (12)	8 (19)	8 (10)	9 (20)	9 (15)	-	0.70[a]	0.65[a]	
**CPS frequency**									
⩽1 per month	17	10	13	14	20	3	0.66[c]	0.93[c]	0.06[c]
2–4 times per month	5	6	5	6	6	5			
>4 times per month	20	15	18	17	28	11			
**HS**									
With HS	22	14	36	0	27	9	0.638[c]	-	0.84[c]
Without HS	20	17	0	37	27	10			
**Number of ASMs**									
1	13	12	10	15	19	6	0.53[c]	0.10[c]	1.00[c]
2	26	15	20	21	30	11			
3	3	4	6	1	5	2			
**Neuropsychology**									
MoCA, mean (SD)	23 (6)	22 (5)	22 (6)	23 (5)	22 (5)	23 (4)	<0.001[b1, b2]	<0.001[b3, b4]	<0.001[b5, b6]
DS-F, mean (SD)	7 (1)	6 (1)	7 (1)	7 (1)	7 (2)	7 (1)	<0.01[b1, b2]	<0.001[b3, b4]	<0.05[b5, b6]
DS-B, mean (SD)	4 (2)	4 (1)	4 (1)	4 (1)	4 (2)	4 (1)	<0.001[b1, b2]	<0.001[b3, b4]	<0.001[b5, b6]
BDT, mean (SD)	33 (10)	29 (8)	32 (10)	32 (9)	31 (9)	33 (10)	<0.001[b2]	<0.05[b3, b4]	<0.01[b5]
TMT A, mean (SD)	50 (24)	56(24)	55 (25)	50 (23)	52 (24)	52 (26)	<0.05[b1, b2]	<0.01[b3]	<0.01[b5]
TMT B, mean (SD)	141 (72)	137 (74)	164 (87)	117 (48)	146 (77)	119 (57)	<0.01[b1, b2]	<0.05[b3, b4]	<0.001[b5]

a obtained by two-sample two-tailed t-test; b obtained by one-way analysis of variance ; b1 post-hoc tests between the LTLE and HC (Tukey HSD test); b2 post-hoc tests between the RTLE and HC (Tukey HSD test); b3 post-hoc tests between the TLE-HS and HC (Tukey HSD test); b4 post-hoc tests between the TLE-nonHS and HC (Tukey HSD test) ; b5 post-hoc tests between the TLE-SGS and HC (Tukey HSD test); b6 post-hoc tests between the TLE-nonSGS and HC (Tukey HSD test); c obtained by chi-square for two or more groups

2 Independent component analysis for cortical network identification

Methods: Spatial independent component analysis (ICA) was performed on smoothing data using an established pipeline by GIFT toolbox. Independent components were calculated using the Infomax and Icasso(Correa et al., 2007). The number of independent components was set at 20 to avoid the network split(Smith et al., 2009; Gürsel et al., 2020). Components were sorted using a semi-manual process by masks included in the GIFT program. Consistent with previous research, we observed the superior frontal cortex was involved in the dorsal attention network(Fox et al., 2006; Ruiz-Rizzo et al., 2018), and the calcarine region was involved in the dorsal attention and visual network(Ruiz-Rizzo et al., 2018).

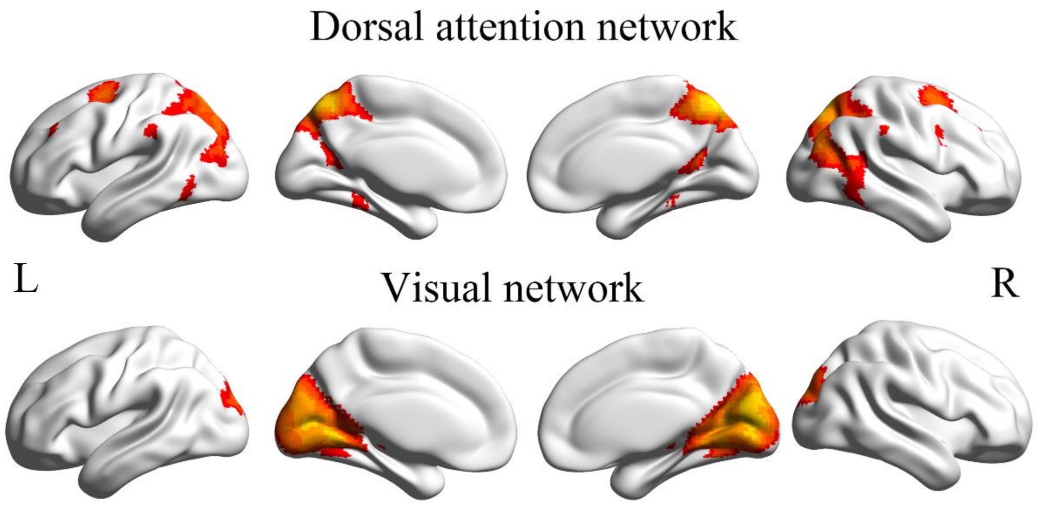

Supplementary Fig 1: Presentation of dorsal attention network and visual network.

References

Correa, N., Adali, T., and Calhoun, V. D. (2007). Performance of blind source separation algorithms for fMRI analysis using a group ICA method. *Magn. Reson. Imaging* 25, 684–694.

Fox, M. D., Corbetta, M., Snyder, A. Z., Vincent, J. L., and Raichle, M. E. (2006). Spontaneous neuronal activity distinguishes human dorsal and ventral attention systems. in *Proceedings of the National Academy of Sciences of the United States of America*, 10046–10051.

Gürsel, D. A., Reinholz, L., Bremer, B., Schmitz-Koep, B., Franzmeier, N., Avram, M., et al. (2020). Frontoparietal and salience network alterations in obsessive–compulsive disorder: insights from independent component and sliding time window analyses. *J. Psychiatry Neurosci. JPN* 45, 214–221.

Ruiz-Rizzo, A. L., Neitzel, J., Müller, H. J., Sorg, C., and Finke, K. (2018). Distinctive Correspondence Between Separable Visual Attention Functions and Intrinsic Brain Networks. *Front. Hum. Neurosci.* 12.

Smith, S. M., Fox, P. T., Miller, K. L., Glahn, D. C., Fox, P. M., Mackay, C. E., et al. (2009). Correspondence of the brain's functional architecture during activation and rest. in *Proceedings of the National Academy of Sciences of the United States of America*, 13040–13045.

3 Cerebellar volume reduction in TLE patients

Supplementary Table 2: Cerebellar volume reduction in TLE patients

Cluster-name	Cluster size	MNI			T value
		x	y	z	
Left_CrusI	677	-39	-74	-31	4.30
Right_CrusI	55	35	-65	-41	3.61
Left_CrusII	114	-36	-59	-42	3.57
Right_CrusII	591	22	-75	-45	3.96
Left_VIIb	585	-24	-66	-48	3.85
Right_VIIb	211	23	-73	-46	3.80
Left_VIIIa	63	-22	-66	-48	3.81
Left_VIIIb	49	-19	-45	-51	3.60
Right_VIIIb	97	11	-51	-61	3.71

Clusters showing significant volume reduction in TLE patients compared to controls (FDR correction $q < 0.05$, cluster $\geqslant 10$).

Cluster size is displayed by voxel numbers.

Coordinates refer to the MNI space coordinates.

Supplementary Table 3: Comparisons of GMV of cerebellar substructures between patients and controls.

Substructure according to SUIT atlas	All TLE	Controls	p-value	FDR q value
Left_I_IV	3.228±0.314	3.345±0.314	0.028	0.047*
Right_I_IV	3.722±0.340	3.839±0.353	0.045	0.058
Left_V	4.231±0.443	4.375±0.432	0.066	0.077
Right_V	4.122±0.408	4.266±0.393	0.032	0.050
Left_VI	9.160±0.997	9.406±0.923	0.205	0.213
Vermis_VI	1.834±0.188	1.905±0.184	0.017	0.032*
Right_VI	7.954±0.815	8.232±0.803	0.047	0.058
Left_CrusI	13.094±1.309	13.736±1.233	0.001	0.028*
Vermis_CrusI	0.017±0.003	0.018±0.004	0.107	0.120
Right_CrusI	12.716±1.361	13.171±1.255	0.043	0.057
Left_CrusII	9.945±1.007	10.391±1.039	0.011	0.028*
Vermis_CrusII	0.403±0.043	0.422±0.043	0.010	0.028*
Right_CrusII	9.295±1.014	9.678±1.017	0.033	0.049*
Left_VIIb	4.999±0.537	5.261±0.555	0.004	0.028*
Vermis_VIIb	0.183±0.022	0.193±0.025	0.011	0.026*
Right_VIIb	4.985±0.552	5.248±0.576	0.006	0.028*
Left_VIIIa	5.256±0.631	5.544±0.600	0.004	0.023*
Vermis_VIIIa	1.134±0.138	1.185±0.136	0.039	0.055
Right_VIIIa	4.779±0.595	5.022±0.584	0.016	0.032*
Left_VIIIb	4.263±0.516	4.511±0.469	0.003	0.042*
Vermis_VIIIb	0.566±0.066	0.596±0.064	0.009	0.028*
Right_VIIIb	4.032±0.478	4.245±0.448	0.006	0.024*
Left_IX	3.282±0.408	3.445±0.406	0.026	0.046*
Vermis_IX	0.677±0.0793	0.717±0.073	0.003	0.028*
Right_IX	3.419±0.415	3.608±0.407	0.008	0.028*
Left_X	0.680±0.068	0.710±0.067	0.013	0.028*
Vermis_X	0.414±0.067	0.416±0.065	0.595	0.595
Right_X	0.652±0.068	0.673±0.077	0.131	0.142

Starred figures (*) are statistically significant after FDR correction for multiple testing.

4 Correlation Analyses of Altered Imaging Measures and Clinical Features

Supplementary Table 4: Correlations of altered imaging measures with clinical features.

	**Age at onset**		**Duration (>10)**		**Number of AED**		**FBTCS frequency**		**MOCA**		TMTA		**TMTB**		DS-B		DS-F		BDT	
	p value	R	p value	R	p value	R	p value	R	p value	R	p value	R	p value	R	p value	R	p value	R	p value	R
CrusI (L)	0.187	0.158	0.977	0.006	0.522	-0.077	0.614	-0.072	0.816	0.030	0.927	-0.012	**0.030**	**-0.298**	0.819	0.029	0.262	0.145	0.855	0.023
CrusII (L)	0.151	0.172	0.552	0.120	0.235	-0.143	0.074	-0.250	0.273	0.141	0.586	-0.070	0.068	-0.253	0.743	-0.042	0.515	0.084	0.786	-0.035
CrusII (R)	0.270	0.133	0.431	0.158	0.304	-0.124	0.072	-0.251	0.252	0.148	0.751	-0.041	**0.024**	**-0.310**	0.712	-0.047	0.451	0.098	0.495	-0.088
VIIb (L)	0.088	0.204	0.655	0.090	0.157	-0.170	0.077	-0.247	0.292	0.136	0.765	-0.038	0.051	-0.270	0.909	-0.015	0.533	0.081	0.884	-0.019
VIIb (R)	0.267	0.134	0.662	0.088	0.181	-0.161	0.106	-0.227	0.124	0.197	0.905	-0.015	0.223	-0.170	0.614	-0.060	0.752	0.041	0.435	-0.100
VIIIa (L)	0.109	0.192	0.740	0.067	0.073	-0.214	**0.043**	**-0.281**	0.224	0.157	0.509	-0.085	**0.017**	**-0.326**	0.873	0.021	0.330	0.126	0.962	0.006
VIIIb (L)	0.193	0.156	0.973	0.007	0.195	-0.156	0.095	-0.234	0.276	0.140	0.487	-0.089	**0.033**	**-0.294**	0.878	-0.020	0.314	0.130	0.931	0.011
VIIIb (R)	0.145	0.175	0.697	-0.079	0.075	-0.213	0.171	-0.193	0.694	0.051	0.441	-0.099	0.101	-0.227	0.824	-0.029	0.572	0.073	0.896	0.017
ICP AD (R)	0.132	-0.181	0.332	-0.194	0.614	-0.061	0.525	-0.090	0.136	-0.191	0.574	-0.072	0.327	0.137	0.817	-0.030	0.383	-0.113	0.362	0.117
ICP AD (L)	0.122	-0.185	0.769	0.059	0.113	-0.190	0.060	-0.263	0.160	-0.180	0.430	-0.101	0.979	0.004	0.285	-0.137	0.504	-0.087	0.845	-0.025
MCP AD (L)	0.383	-0.105	0.338	-0.184	0.205	-0.152	0.412	-0.116	0.364	-0.117	0.717	-0.047	0.183	0.186	0.933	-0.011	0.108	-0.206	0.808	0.031
FC (right crus II and left parahippocampal cortex)	0.970	0.005	0.387	0.185	0.761	-0.039	0.572	0.085	0.418	0.110	**0.003**	**-0.360***	0.448	-0.113	**0.043**	**0.256**	0.390	0.117	**0.009**	**0.325**
FC (left VI and right superior frontal gyrus)	0.822	-0.029	0.223	0.258	0.497	0.087	0.429	0.118	0.965	0.006	0.544	0.078	0.675	0.063	0.481	-0.090	0.613	-0.069	**0.028**	**-0.277**
FC (left VIIb and left calcarine)	0.277	-0.139	**0.034**	**0.433**	0.606	-0.066	0.499	0.101	0.906	0.016	0.454	0.096	0.858	0.027	0.997	0.001	0.556	-0.080	0.321	-0.127
FC (left VIIIa and left calcarine)	0.341	-0.122	**0.003**	**0.560***	0.657	0.057	0.632	0.072	0.298	0.142	0.791	-0.034	0.828	0.033	0.959	0.007	0.774	-0.039	0.083	-0.220
FC (left VIIIb and bilateral calcarine)	0.876	0.020	0.114	0.231	0.792	0.034	0.639	0.863	0.614	-0.069	0.072	-0.228	0.642	-0.070	0.883	-0.019	0.900	-0.017	**0.028**	**-0.277**

Starred figures (*) are statistically significant after FDR correction for multiple testing. In bold are results with uncorrected p <0.05.

5 Subgroup analysis comparing RTLE, LTLE, and HC

5.1 Volume feature

Compared to controls, LTLE patients showed gray matter atrophy mainly in left crus I, right crus II, bilateral red nucleus, right mesencephalic reticular formation and periaqueductal gray.

Compared to controls, RTLE patients also showed gray matter reduction in left crus I, bilateral red nucleus, mesencephalic reticular formation, and periaqueductal gray (Supplementary Fig 2 and table 5).

5.2 DTI feature

Compared to controls, LTLE patients showed decreased FA in the right red nucleus and mesencephalic reticular formation, and areas around bilateral lobules IV and V. In addition, decreased AD in the right red nucleus, left mesencephalic reticular formation, left MCP, and left inferior olivary nucleus were also observed.

Compared to controls, RTLE patients showed decreased FA and AD in the right red nucleus (Supplementary Fig 2).

Tract analysis showed AD reduction in left MCP and right ICP in LTLE patients compared to controls (Supplementary table 6). Previous studies suggested that LTLE patients appear to have a greater degree of white matter atrophy and dysconnectivity relative to RTLE patients, which might result from brain asymmetry (Zhao et al., Frontiers in neurology, 2019).

5.3 FC feature

Similar FC alterations were found in comparisons between LTLE/RTLE and HC (Supplementary Fig 3).

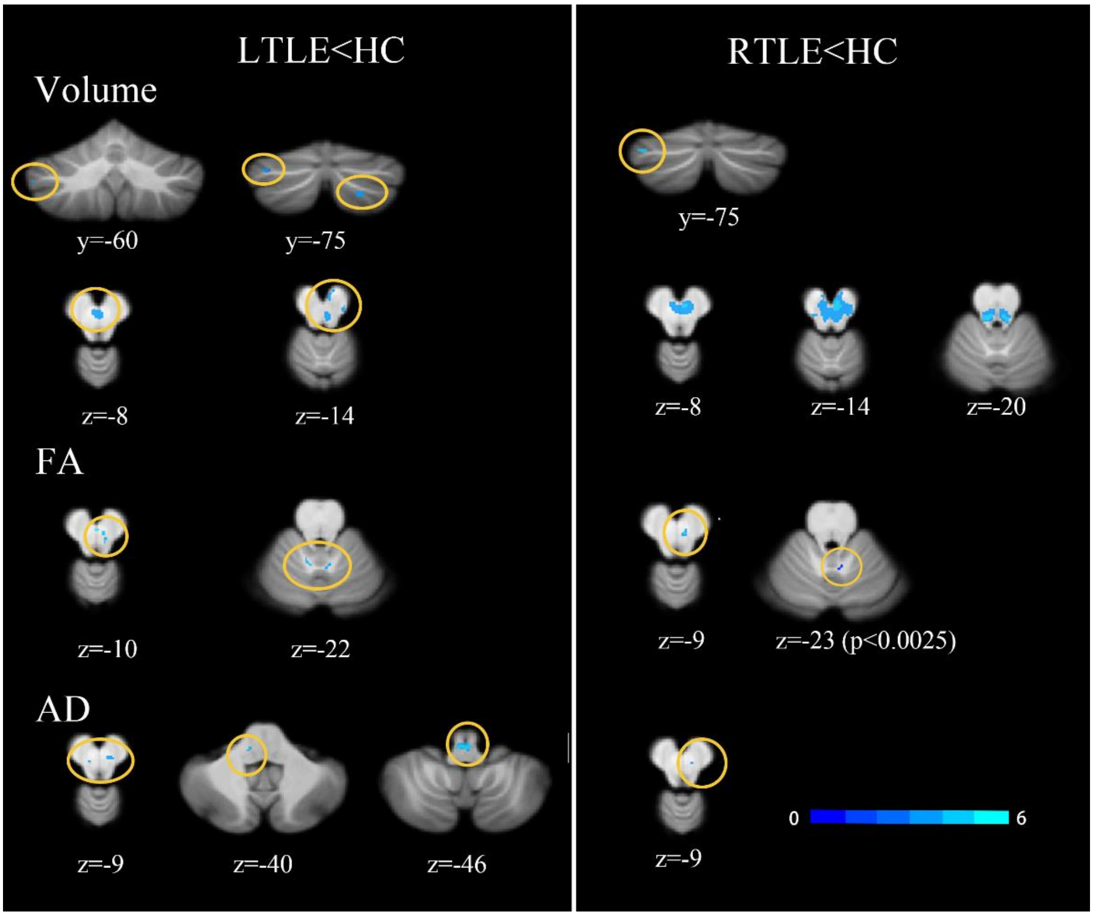

Supplementary Fig 2: Structural alterations in patients with different lateralization FWE correction $p< 0.05$, cluster ⩾ 10. Results are projected on the SUIT atlas.

Supplementary Table 5: Cerebellar lobule atrophy in patients with different lateralization

cluster-name	cluster-size	MNI			T value
		x	y	z	
**LTLE<HC**					
Left_CrusI	50	-52	-59	-39	3.44
Right_CrusII	110	28	-79	-47	3.82
**RTLE< HC**					
Left_CrusI	209	-40	-74	-31	3.78

Clusters showing significant volume reduction (FWE correction $p < 0.05$, cluster ⩾ 10).

Cluster size is displayed by voxel numbers.

Coordinates refer to the MNI space coordinates.

Supplementary Table 6: Tract analyses results in patients with different lateralization

P-value	LTLE vs HC	RTLE vs HC	LTLE vs RTLE
**AD**			
L-MCP	0.014*	0.650	0.706
L-ICP	0.127	0.114	1.000
R-ICP	0.008*	0.183	1.000

*Significant results similar to whole group comparisons (Bonferroni test, $p< 0.05$).

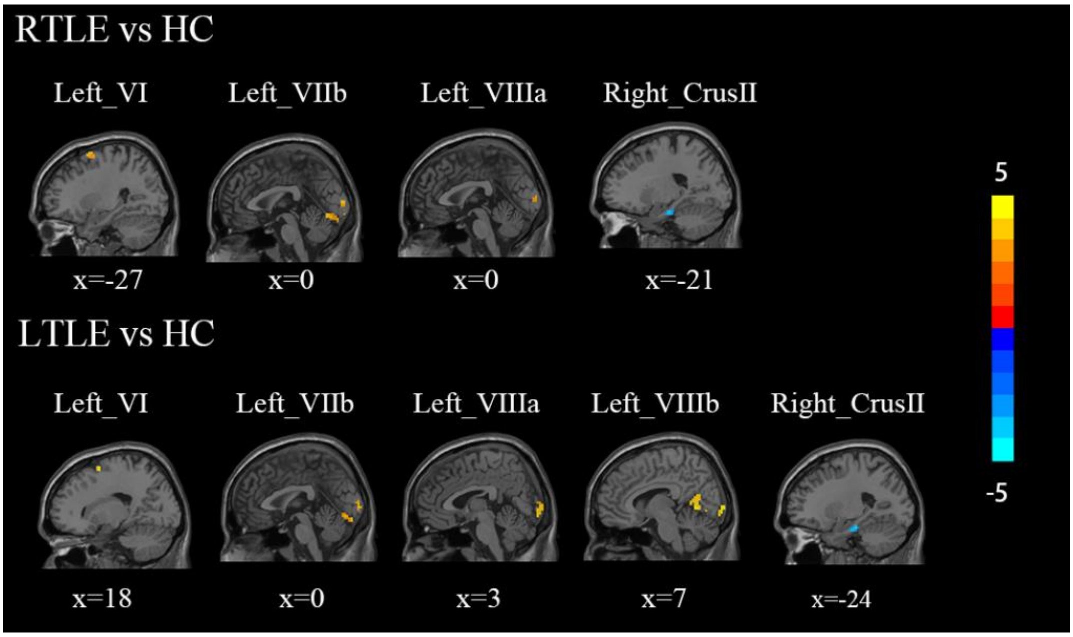

Supplementary Fig 3: Functional alterations in patients with different lateralization
FWE correction $p < 0.05$, cluster ⩾ 10

6 Subgroup analysis comparing TLE-HS, TLE-nonHS, and HC

6.1 Volume feature

Compared to controls, TLE-HS patients showed gray matter atrophy in left crus I, VIIb, VIIIa, VIIIb, and right crus II. Atrophy in bilateral red nuclei, mesencephalic reticular formation and periaqueductal gray were also observed.
Post hoc analysis showed no differences between TLE-nonHS and HC (Supplementary Fig 4 and table 7).

6.2 DTI feature

Compared to controls, TLE-HS patients showed decreased FA in right red nucleus, mesencephalic reticular formation, and cluster around bilateral lobules IV and V. AD reduction in the right red nucleus, bilateral mesencephalic reticular formation, left MCP, right ICP, and left inferior olivary nucleus were also found.
Compared to controls, TLE-nonHS also showed decreased FA in right red nucleus and cluster around right lobules IV and V.
We found that TLE-HS had a decreased FA in the left inferior olivary nucleus than TLE-nonHS patients (Supplementary Fig 4).
Tract analysis showed decreased AD in left MCP and bilateral ICP in TLE-HS patients compared to controls (Supplementary table 8).

6.3 FC feature

Similar FC alterations were found in comparisons between TLE-HS/TLE-nonHS and HC (Supplementary Fig 5).

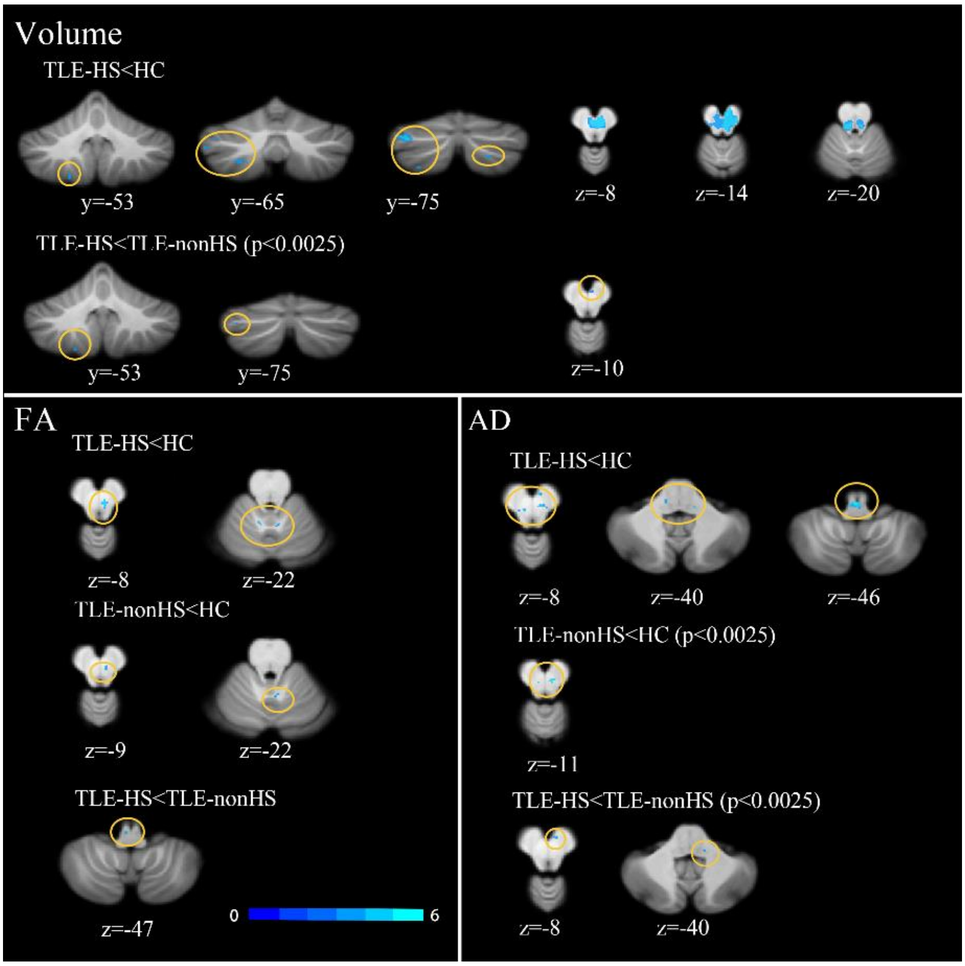

Supplementary Fig 4: Structural alterations in patients with or without HS

Clusters showing significant volume reduction (FWE correction $p < 0.05$, cluster ⩾ 10).
The exploratory threshold was set at $p < 0.05$, uncorrected with cluster ⩾ 10(Limbachia et al., 2021). Results are projected on the SUIT atlas.

Supplementary Table 7: Structural alterations in patients with or without HS

cluster-name	cluster-size	MNI			T value
		x	y	z	
**TLE-HS<HC**					
Left_CrusI	436	-42	-71	-31	4.29
Right_CrusII	16	22	-76	-45	3.20
Left_VIIb	77	-23	-66	-48	3.86
Left_VIIIa	11	-22	-66	-48	3.84
Left_VIIIb	24	-19	-52	-58	3.91
**TLE-HS< TLE-nonHS** (p<0.0025)					
Left_CrusI	10	-38	-75	-34	3.12
Left_VIIIb	10	-19	-53	-60	3.26

FWE correction $p < 0.05$, cluster ⩾ 10.
An exploratory threshold was set at $p < 0.05$, uncorrected with cluster ⩾ 10(Limbachia et al., 2021). Cluster size is displayed by voxel numbers.
Coordinates refer to the MNI space coordinates.

Supplementary Table 8: Tract analyses results in patients with or without HS

P-value	TLE-HS vs HC	TLE-nonHS vs HC	TLE-HS vs TLE-nonHS
**AD**			
L-MCP	0.032*	0.267	1.000
L-ICP	0.015*	0.584	0.548
R-ICP	0.011*	0.106	1.000

*Significant results similar to whole group comparisons (Bonferroni test, $p < 0.05$).

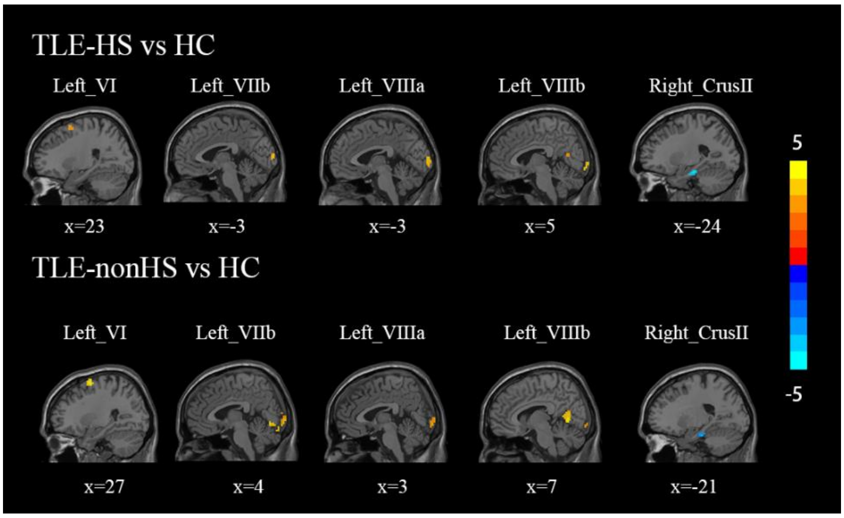

Supplementary Fig 5: Functional alterations in patients with or without HS

FWE correction $p < 0.05$, cluster ⩾ 10

7 Subgroup analysis comparing TLE-SGS, TLE-nonSGS, and HC

7.1 Volume feature

Compared to controls, TLE-SGS showed cerebellar atrophy in widespread cerebellar lobules, including bilateral crus I, crus II, VIIb, VIIIa, VIIIb, and IX. Atrophy in bilateral red nuclei, mesencephalic reticular formation, periaqueductal gray and cerebellar afferent pathways was also observed.

Compared to TLE-nonSGS, TLE-SGS also showed decreased volume in left crus I, bilateral mesencephalic reticular formation, and cerebellar afferent pathways.

There were no significant volume differences between TLE-nonSGS and controls (Supplementary Fig 6 and table 9).

7.2 DTI feature

Compared to controls, decreased FA and AD were observed in the right red nucleus in the TLE-SGS group. TLE-SGS also showed FA reduction around right lobule IV and V (Supplementary Fig 6).

Tract analysis showed decreased AD in bilateral ICP in TLE-SGS patients. TLE-nonSGS only showed decreased AD in left MCP (Supplementary table 10).

7.3 FC feature

Similar FC alterations were found in comparisons between TLE-SGS/TLE-nonSGS and HC (Supplementary Fig 7).

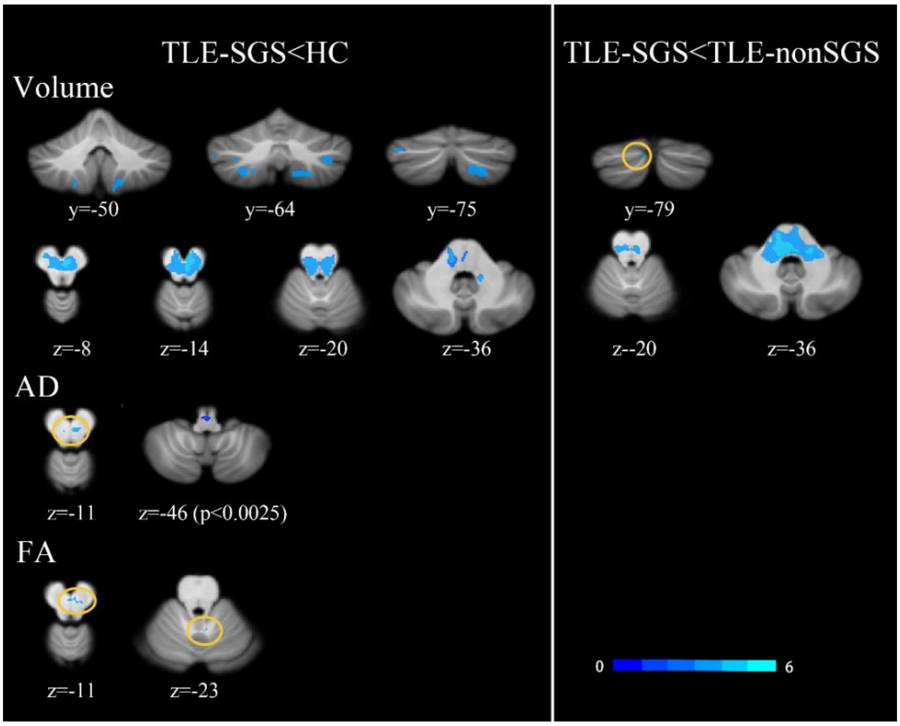

Supplementary Fig. 6: Cerebellar volume reduction in patients with or without SGS

Clusters showing significant volume reduction (FWE correction $p < 0.05$, cluster ⩾ 10).

Results are projected on the SUIT atlas.

Supplementary Table 9: Structural alterations in patients with or without SGS

cluster-name	cluster-size	MNI			T value
		x	y	z	
**TLE-SGS<HC**					
Left_CrusI	354	-38	-57	-41	4.036629
Right_CrusI	268	35	-66	-40	4.15523
Left_CrusII	223	-38	-56	-43	3.864569
Right_CrusII	822	21	-76	-45	4.264679
Left_VIIb	532	-24	-67	-48	4.183636
Right_VIIb	611	20	-74	-46	4.187293
Left_VIIIa	138	-22	-66	-48	4.096055
Right_VIIIa	477	18	-68	-50	3.796458
Left_VIIIb	253	-19	-45	-51	4.239696
Right_VIIIb	396	18	-51	-55	4.148006
Left_IX	15	-13	-44	-50	3.7925
Right_IX	45	11	-51	-58	3.929296
**TLE-SGS< TLE-nonSGS**					
Left_CrusI	13	-6	-79	-29	4.626584

FWE correction $p < 0.05$, cluster ⩾ 10.
Coordinates refer to the MNI space coordinates.

Supplementary Table 10: Tract analyses results in patients with or without SGS

P-value	TLE-SGS vs HC	TLE-nonSGS vs HC	TLE-SGS vs TLE-nonSGS
**AD**			
L-MCP	0.090	0.100	1.000
L-ICP	0.066	0.302	1.000
R-ICP	0.016*	0.116	1.000

*Significant results similar to whole group comparisons (Bonferroni test, $p < 0.05$).

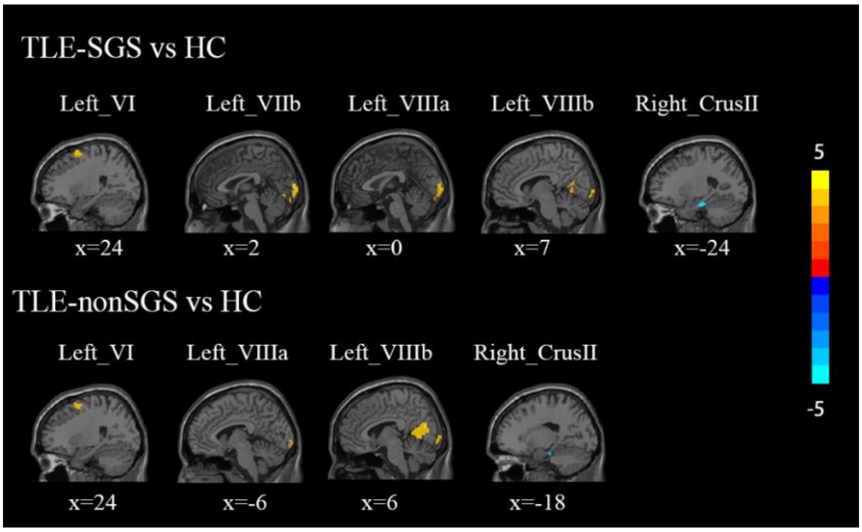

Supplementary Fig 7: Functional alterations in patients with or without SGS

FWE correction $p < 0.05$, cluster ⩾ 10

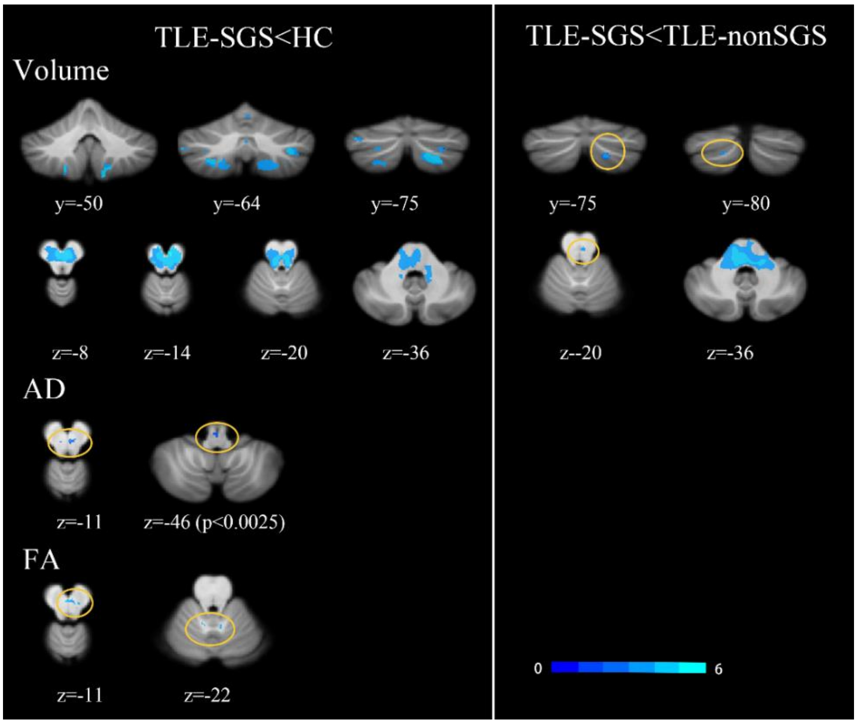

Supplementary Fig. 8: Cerebellar volume reduction in patients with or without SGS using nonparametric testing

Clusters showing significant volume reduction (FWE correction $p < 0.05$, cluster ⩾ 10).

Results are projected on the SUIT atlas.

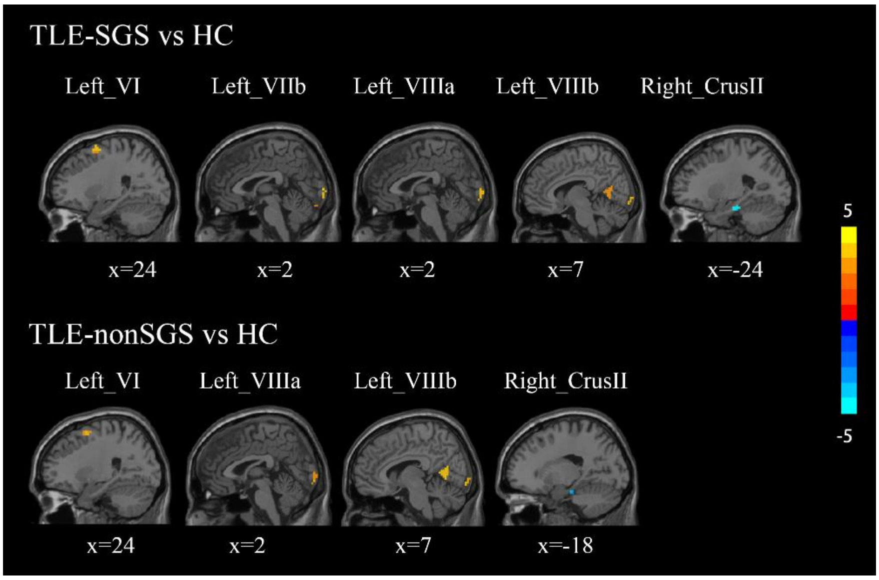

Supplementary Fig 9: Functional alterations in patients with or without SGS using nonparametric testing

FWE correction $p < 0.05$, cluster ⩾ 10
